# Rational design of a nanoparticle platform for oral prophylactic immunotherapy to prevent immunogenicity of therapeutic proteins

**DOI:** 10.1038/s41598-021-97333-0

**Published:** 2021-09-08

**Authors:** Nhan H. Nguyen, Fiona Y. Glassman, Robert K. Dingman, Gautam N. Shenoy, Elizabeth A. Wohlfert, Jason G. Kay, Richard B. Bankert, Sathy V. Balu-Iyer

**Affiliations:** 1 Department of Pharmaceutical Sciences, School of Pharmacy and Pharmaceutical Sciences, SUNY-University at Buffalo, 359 Pharmacy Building, Buffalo, NY 14214 USA; 2 Department of Microbiology and Immunology, Jacobs School of Medicine and Biomedical Sciences, SUNY-University at Buffalo, Buffalo, NY USA; 3 Department of Oral Biology, School of Dental Medicine, SUNY-University at Buffalo, Buffalo, NY USA; 4 Present Address: CSL Behring, King of Prussia, PA USA; 5 Present Address: Regeneron Pharmaceuticals, Tarrytown, NY USA

**Keywords:** Nanoparticles, Immunotherapy, Phospholipids

## Abstract

The safety and efficacy of several life-saving therapeutic proteins are compromised due to their immunogenicity. Once a sustained immune response against a protein-based therapy is established, clinical options that are safe and cost-effective become limited. Prevention of immunogenicity of therapeutic proteins prior to their initial use is critical as it is often difficult to reverse an established immune response. Here, we discuss a rational design and testing of a phosphatidylserine-containing nanoparticle platform for novel oral prophylactic reverse vaccination approach, i.e., pre-treatment of a therapeutic protein in the presence of nanoparticles to prevent immunogenicity of protein therapies.

## Introduction

Therapeutic proteins are one of the fastest growing class of drugs, as they offer treatment options for several diseases with minimal off-target effects. However, the unwanted immune responses against these life-saving therapies not only impacts their safety profile but also efficacy^1–3^. For example, about 89–100% of Pompe disease patients who receive recombinant acid alpha glucosidase (GAA) as enzyme replacement therapy develop anti-GAA antibodies^4,5^. Once a sustained immune response is established, the efficacy of this life-saving therapy is compromised and tolerance inducing regimens using bortezomib in combination with rituximab, methotrexate, and intravenous immunoglobulin are attempted to rescue these high titer patients^6^. However, the use of such immunosuppressive agents poses a risk for secondary infections. In the case of Hemophilia A (HA), a bleeding disorder, about one third of the severe HA patients receiving replacement therapy using recombinant Factor VIII (FVIII) develop neutralizing anti-FVIII antibodies, referred to as inhibitors, that abrogate the biological activity and hemostatic efficacy of the administered FVIII^7–9^. Clinical options after the development of antibodies are very costly (over $700,000/year/patient) and, in some patients, ineffective^10^. Therefore, strategies to prevent unwanted immune responses against therapeutic proteins are desirable for clinical management, patient care, and cutting health care costs.

Our previous studies have shown that phosphatidylserine (PS) can convert an immunogen to a tolerogen, thereby leading to the reduction of unwanted immune responses. The subcutaneous administration of FVIII and GAA in the presence of nanoparticles containing double-chain PS species were able to induce immunological hypo-responsiveness in relevant mouse models^11–14^. Furthermore, in vitro studies demonstrated that PS promotes phenotypic changes in dendritic cells (DCs) and converts them to the tolerogenic type^15^. Surprisingly when given via oral route, nanoparticles containing double-chain PS did not induce tolerance to the same proteins as seen with subcutaneous administration. This is possibly due to the uptake and mechanistic differences of oral tolerance compared to other routes of administration. PS externalization to the outer leaflet of bilayer membrane and PS surface density are critical determinants promoting differential recognition by immune-regulatory PS receptors including T-cell immunoglobulin and mucin domain containing 4 (TIM-4)^16,17^. Additionally, the uptake of PS-containing particles across the intestinal wall is postulated to be facilitated by microfold (M) cells, which predominantly express the scavenger receptor class B type 1 (SR-B1), clusterin, and annexin V^18^. These proteins have been demonstrated to bind to and/or interact with PS exposed on the outer leaflet of apoptotic cells or PS-containing liposomes, which aids the phagocytosis and internalization of cells or vesicles expressing PS^19–22^. Therefore, we rationally designed and tested a nanoparticle platform with the optimal structural (single-acyl chain lysophosphatidylserine [Lyso-PS] derivative) and biophysical characteristics, including size and PS surface exposure. This platform serves as a tolerogenic form of protein for a novel lipid-based immunotherapy to deliver proteins in the presence of PS to prevent their immunogenicity. The rationale for this approach is that pre-exposure of a protein in the presence of PS induces tolerance and blocks the patients’ immune response towards that protein prior to the initiation of the therapy. The utilization of oral administration would also allow for the exposure of formulations to the mucosal immune system, which has evolved as an effective, default tolerogenic site^23,24^. Furthermore, oral administration can offer clinical translatability due to the simplicity, convenience, and high patient compliance.

## Results

### Design of lipid nanoparticles for immunotherapy

PS is an anionic phospholipid that is generally present in the inner leaflet of a healthy cell, but flips to the outer leaflet when cells undergo apoptosis^25,26^. This externalization of PS sends an “eat me” signal to phagocytes for a clearance of cell debris in an immunologically silent manner, therefore “maintaining” tolerance towards self-proteins by immunological ignorance termed as a “tolerate/ignore me” signal^27^. However, our efforts to reduce immunogenicity of therapeutic proteins demonstrated that the externalization of PS promotes an active learning process through induction of antigen-specific tolerance^14^. It has been identified that TIM-4 is one of the PS receptors, which is exclusively expressed on antigen presenting cells (APCs)^28–30^. Studies from our lab have shown that PS-mediated tolerance induction involves the interaction between PS nanoparticles and TIM-4^14^. Interestingly, TIM-4 is sensitive to PS density exposed on the surface of bilayer membrane^16,17^ and mediates differential recognition of apoptotic events over other forms of PS externalization to promotes tolerance^28,31^. Additionally, particle size and PS exposure on the outer leaflet of the particles are critical biophysical parameters for cellular uptake and receptor binding^17,25,32,33^, that in turn influence the biological functions of PS-containing vesicles. Therefore, we designed a nanoparticle containing PS with optimal biophysical characteristics and PS externalization for a more effective tolerance induction. While the structure of PS is generally known to consist of a serine headgroup and two fatty acid acyl chains connected together by a glycerol backbone, several derivatives of PS also exist in which they differ in the lengths of fatty acid acyl chain, degree of unsaturation, and number of acyl chains. The PS derivative with only one fatty acid chain is termed Lyso-PS. Since phosphatidylcholine is the major lipid component providing structural framework of biological membranes, we use dimyristoylphosphatidylcholine (DMPC) as the base phospholipid to form bilayer of the nanoparticles. Therefore, pure DMPC nanoparticles are used as the control. Unlike the structure of PS and Lyso-PS, DMPC contains a net neutral charge with zwitterionic choline headgroup. After testing various formulations comprised of different PS species, we found that DMPC nanoparticles containing single-chain 18:1 Lyso-PS had a significantly smaller mean particle size (112 ± 9.6 nm) compared to pure DMPC (189 ± 24.9 nm) and DMPC containing double-chain brain PS or double-chain 18:1 PS (169 ± 9.3 nm and 169 ± 0.4 nm, respectively) despite the identical preparation procedure (thin film method followed by extrusion through 200 nm polycarbonate membranes) (Table 1). The decrease in particle size of Lyso-PS nanoparticles is likely due to the increase in surface curvature of the vesicles, caused by the partitioning of the cone-shaped Lyso-PS into DMPC bilayers. In contrast, the partitioning of cylindrical-shaped double-chain PS into DMPC bilayers increased the fluidity of the nanoparticles, but did not alter the particle curvature or size.

**Table 1:** Particle size and size distribution of nanoparticles containing different PS species varying in the number of acyl chains.

Lipid composition	Mean particle size ± SD (nm)	Polydispersity ± SD
DMPC (100%)	189 ± 24.9	0.17 ± 0.02
Brain PS (30%)	169 ± 9.3	0.15 ± 0.03
18:1 PS (30%)	169 ± 0.4	0.13 ± 0.03
18:1 Lyso-PS (30%)	112 ± 9.6*^†‡^	0.10 ± 0.02

One-way ANOVA followed by Tukey’s post-hoc multiple comparison test were performed to detect significant differences (*P* < 0.05).

*Significant difference from DMPC.

^†^Significant difference from Brain PS.

^‡^Significant difference from 18:1 PS.

**Figure 1:**
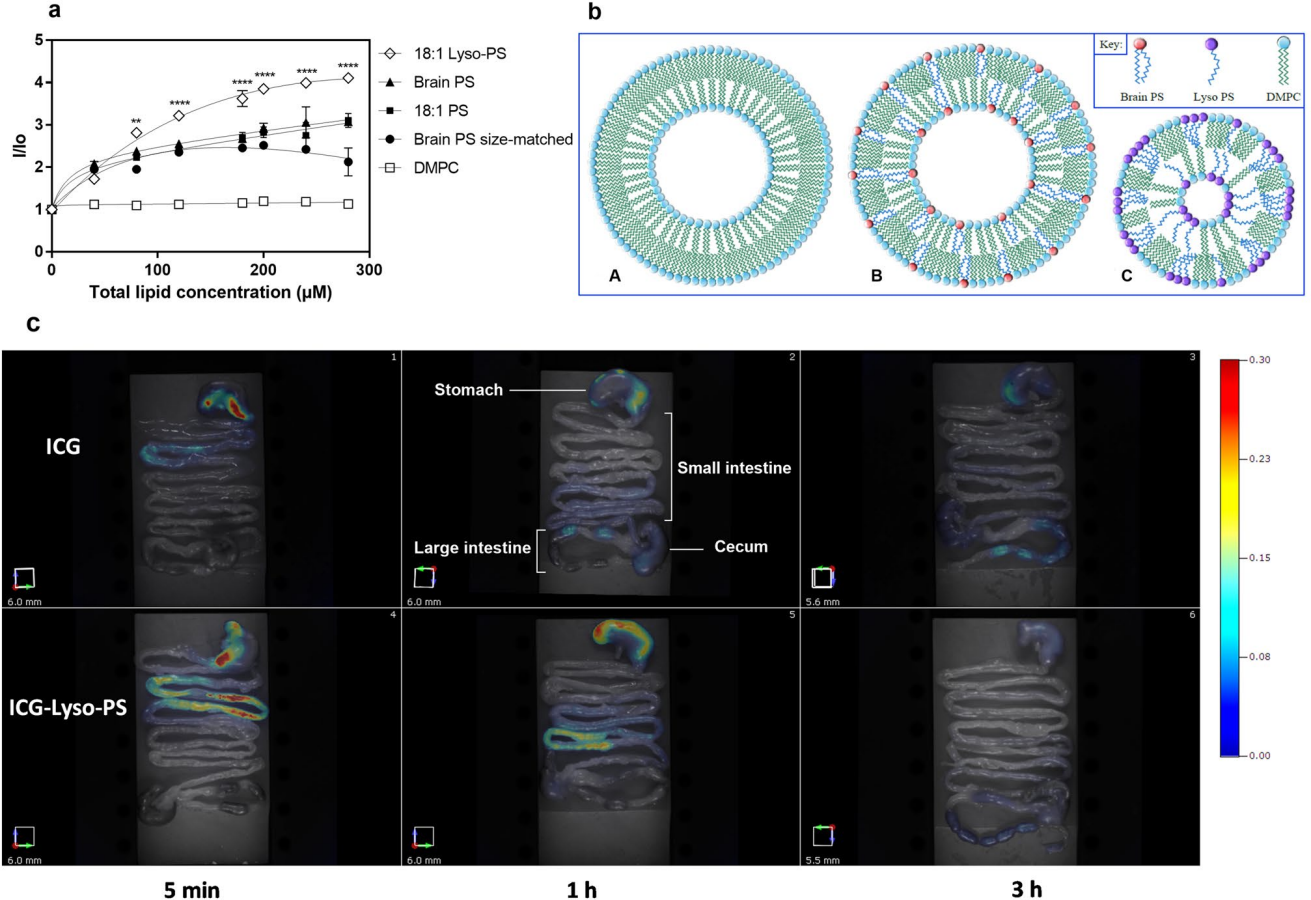
Biophysical characteristics of lipid nanoparticles for immunotherapy. (**a**) Surface exposure of different PS species on the nanoparticles as detected by PSVue fluorescence signal. Changes in fluorescence intensity I/I_0_ were monitored as a function of total lipid concentration and fitted in GraphPad Prism using a single site-total binding model with nonlinear least squares fitting. Data are represented as mean ± SD. Two-way ANOVA followed by Tukey’s post-hoc analysis was performed to detect statistical significance, *P < 0.05. (**b**) Proposed schematic molecular arrangement of Lyso-PS nanoparticles (C) compared to DMPC (A) and PS nanoparticles (B). Mean diameter of Lyso-PS nanoparticles is reduced to reflect the fact that these nanoparticles are significantly smaller than DMPC and PS nanoparticles. (**c**) Stability and disposition of tolerogenic lipid particles in the GI tract following oral administration detected by near infrared fluorescence imaging. Representative graphs from 3 independent experiments were shown.

To examine the exposure of PS on the surface of nanoparticles containing different PS species, a titration study was conducted using the PS-binding fluorescent probe PSvue 550. Similar to annexin V, PSvue binds selectively with high affinity to anionic phospholipids, especially PS, that are present on the surface of vesicles or cell membranes by crosslinking with the phosphate group and produces strong emission intensity upon binding^34,35^. To evaluate the exposure of PS on the particle surface, change in fluorescence intensity was monitored as a function of total lipid concentration (Fig. 1a). As expected, no change in fluorescence signal was observed upon the addition of PSvue to DMPC nanoparticles, while all PS-containing ones displayed strong emission intensity. Lyso-PS nanoparticles exhibited the highest change in fluorescence intensity compared to double-chain PS, suggesting a much higher distribution of Lyso-PS on the outer leaflet of these vesicles. To determine whether particle size contributed to PS surface density, nanoparticles containing double-chain PS that were size-matched to Lyso-PS nanoparticles were also examined. The results showed that irrespective of particle size, PS exposure was much higher for Lyso-PS nanoparticles. This could be due to the formation of non-uniform clusters of single-chain Lyso-PS on the surface of the nanoparticles, whereas double-chain PS distributed more uniformly on the inner and outer layers of the vesicles (Fig. 1b). It is likely that the combined effects of small particle size, high PS surface density, and clustering of Lyso-PS nanoparticles can provide a more available total surface area that is suitable for receptor recognition (for example TIM-4), cellular uptake, and in turn, oral tolerance induction compared to double-chain PS nanoparticles. Additionally, we expected that any strategies which increase the surface exposure and clustering of PS on the surface of the nanoparticles, including changes in compositions, can potentially enhance the nanoparticles’ tolerogenic activity.

In the gastrointestinal (GI) tract, oral tolerance is mediated by antigen exposure to CD103+ DCs. Upon antigen uptake and processing, CD103+ DCs migrate to the mesenteric lymph nodes (MLNs), which are the lymphoid organs draining the gut, where they drive the differentiation of naïve T cells to different types of regulatory T cells (T_regs_) with the help of cytokines, enzymes, and metabolite signaling such as TGF-β, indoleamine 2,3-dioxygenase, and retinoic acid^36,37^. However, the access of therapeutic proteins in their original forms to gut-associated lymphoid tissue (GALT) is challenging due to the harsh conditions of oral delivery. To further understand the fate of Lyso-PS nanoparticles when given orally and their ability to deliver antigens to GALT for tolerance induction, we investigated the stability and distribution of Lyso-PS nanoparticles in vivo. The fluorescence probe indocyanine green (ICG) was associated with the hydrophobic core of Lyso-PS nanoparticles and tracked using detailed organ imaging (Fig. 1c). No fluorescence signal was detectable in untreated mice, indicating the absence of background autofluorescence. Mice receiving aqueous ICG solution were used as the controls, as ICG is an acid-labile imaging agent with the fluorescence being rapidly quenched at low pH environment^38^. The results at 5 min post administration showed that the encapsulation of ICG in Lyso-PS nanoparticles significantly increased the fluorescence signal compared to free ICG in aqueous solution, as observed previously^39,40^. This is because the insertion of ICG into the hydrophobic domain of the nanoparticle stabilizes and enhances its fluorescence properties, as ICG is likely shielded from water which degrades ICG fluorescence over time. Therefore, fluorescence intensity is a direct measure of nanoparticle integrity. At 5 min post administration, both aqueous ICG and ICG-Lyso-PS nanoparticles started to travel down to the small intestine. However, the distribution of ICG-Lyso-PS nanoparticles in the upper GI tract was substantially more extensive. After 1 h, an intense fluorescence signal was still detectable for ICG-Lyso-PS nanoparticles in the small intestine with a delay in transit time compared to aqueous ICG, suggesting that the particles are stable and can protect the cargoes from enzymatic degradation. At 3 h post administration, ICG delivered by Lyso-PS nanoparticles was eliminated from the body through fecal matters. The data are consistent with the fact that Lyso-PS nanoparticles are stable in the harsh environment of the GI tract to protect their cargoes and establish the feasibility for oral immunization to target GALT.

### Lyso-PS nanoparticles prevent immunogenicity in an antigen specific manner

**Figure 2:**
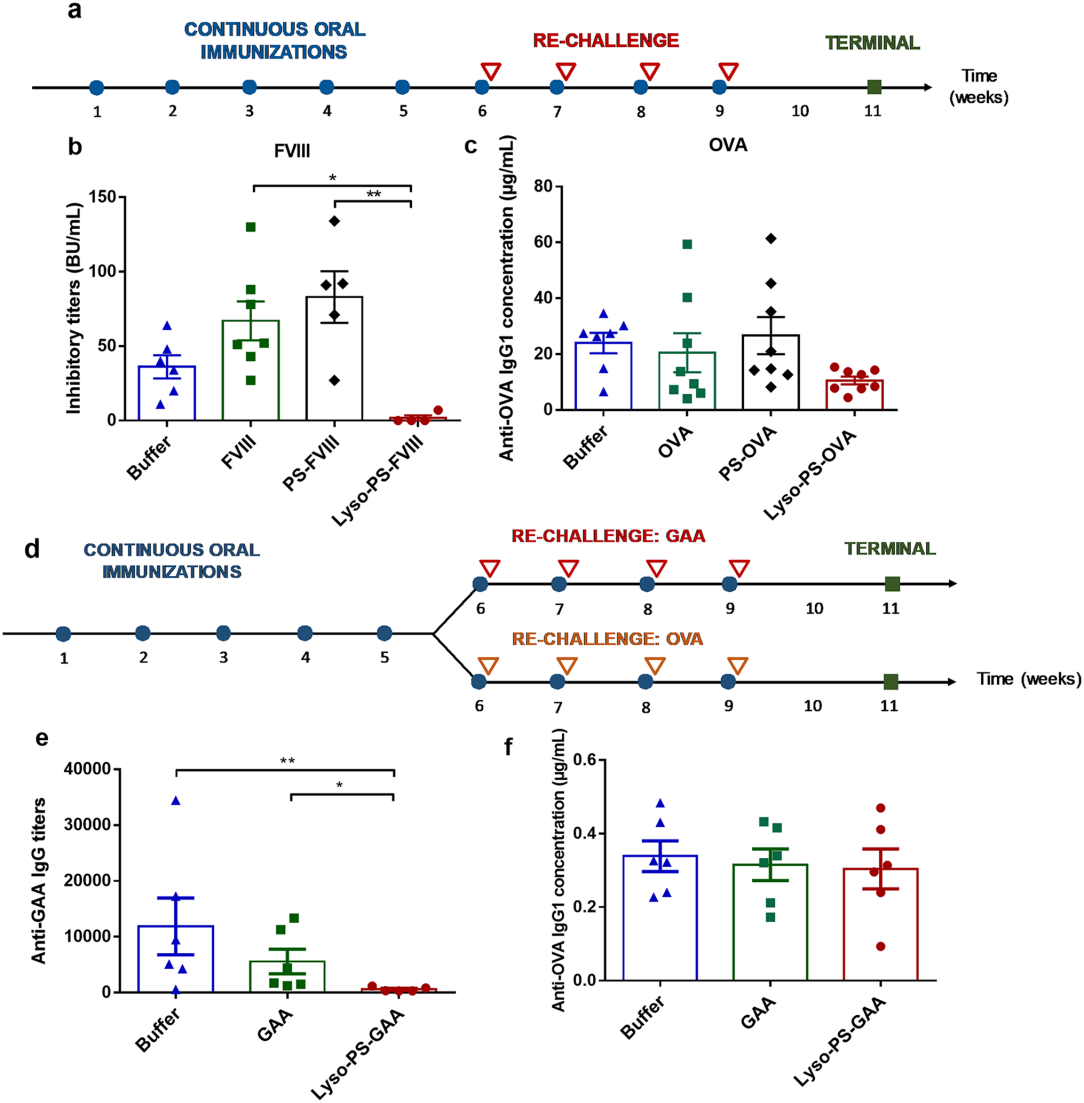
Tolerogenic lipid particles prevents immunogenicity of several antigens in an antigen-specific manner. (**a**) General immunization protocol—oral pre-exposure of antigens in the presence and absence of Lyso-PS nanoparticles, followed by re-challenge with free antigens. (**b**) Tolerogenic lipid particles utilizing Lyso-PS nanoparticles, but not PS ones, induced oral tolerance towards FVIII in HA mice following four weekly re-challenge with 0.4 μg free FVIII, and OVA (**c**) in Swiss Webster mice following four weekly re-challenge with 2 μg free OVA. (**d**) Immunization protocol for antigen specificity study. (**e**) Total anti-GAA antibodies after four weekly re-challenges with 1 μg free GAA. (**f**) Total anti-OVA IgG1 concentration after four weekly re-challenges with 1 μg free OVA. All data are represented as mean ± SEM. Statistical significance was denoted by P < 0.05 (*) by a one-way ANOVA test followed by Tukey’s post-hoc analysis on log-transformed data.

The ability of nanoparticles containing Lyso-PS to prevent immunogenicity in relevant mouse models was next investigated. The general immunization protocol via oral gavage is illustrated in Fig. 2a. As seen in Fig. 2b, mice pre-treated with Lyso-PS-FVIII displayed a statistically significant decrease in inhibitor development (1.8 ± 2 BU/mL) compared to free FVIII pre-treated mice (67.0 ± 13 BU/mL), despite the aggressive re-challenge with free FVIII which mimics the initiation of the clinical therapy. Notably, anti-FVIII inhibitors were not detectable in 75% of the mice pre-treated with Lyso-PS-FVIII. Similar observations that oral pre-exposure with Lyso-PS-FVIII effectively reduced immunogenicity were consistently detected in four independent experiments, using different FVIII preparations including full-length FVIII and long acting FVIII Fc fusion protein. In contrast, animals that were given double-chain PS nanoparticles during the pre-exposure window elicited titers (83.0 ± 17 BU/mL) that are comparable to those receiving buffer and free FVIII. In an independent study evaluating the impacts of particle size on tolerance induction, Lyso-PS nanoparticles induced effective oral tolerance while nanoparticles comprised of double-chain PS with similar particle size (~ 100 nm) did not. Particularly, mice pre-treated with size-matched PS-FVIII nanoparticles as oral pre-exposures developed higher anti-FVIII inhibitory titers compared to those received Lyso-PS-FVIII. This is consistent with our observation that PS surface exposure of Lyso-PS nanoparticles is higher than double-chain PS ones, regardless of particle size. We also found in a separate experiment that 18:1 lysophosphatidylcholine (Lyso-PC) is not effective in inducing tolerance compared to 18:1 Lyso-PS, ruling out the role of 18:1 acyl chain as the major structural determinant. The ability of Lyso-PS (but not double-chain PS) nanoparticles to induce hypo-responsiveness can also be extended and applied to other proteins. Specifically, when the similar approach was tested with ovalbumin (OVA), all treatment groups except Lyso-PS-OVA developed a robust anti-OVA antibody response upon the aggressive subcutaneous re-challenge with free antigen (Fig. 2c, 24.0 ± 3.7 µg/mL for buffer, 20.5 ± 7.0 µg/mL for free OVA, and 26.6 ± 6.7 µg/mL for PS-OVA). In contrast, the majority of mice pre-exposed to Lyso-PS-OVA as oral immunizations consistently displayed substantially lower anti-OVA antibody development (10.6 ± 1.4 µg/mL). In order to further investigate the antigen specificity of Lyso-PS-mediated hypo-responsiveness, B6.129 GAA^−/−^ mice were administered GAA orally in the presence and absence of Lyso-PS nanoparticles. Starting at week 6, half of the animals were re-challenged with free GAA while the other half received free OVA, a secondary non-cross-reactive antigen, and anti-GAA as well as anti-OVA titers were measured (Fig. 2d). If Lyso-PS-mediated tolerance induction is antigen specific, anti-GAA antibody level is expected to be low, while a robust immune response against the unrelated antigen, OVA, should be observed. The results showed that mice receiving Lyso-PS-GAA expressed significantly lower anti-GAA titer level, and the majority of them displayed very little to no response compared to those treated with free GAA and buffer (Fig. 2e). As expected, all animals re-challenged with OVA developed comparable robust anti-OVA antibody response, irrespective of the oral pre-treatments (Fig. 2f). This suggests that oral tolerance induced by Lyso-PS nanoparticles can prevent antibody development in an antigen specific manner and not by non-specific global immunosuppression.

### Mechanism of Lyso-PS-mediated oral tolerance

**Figure 3:**
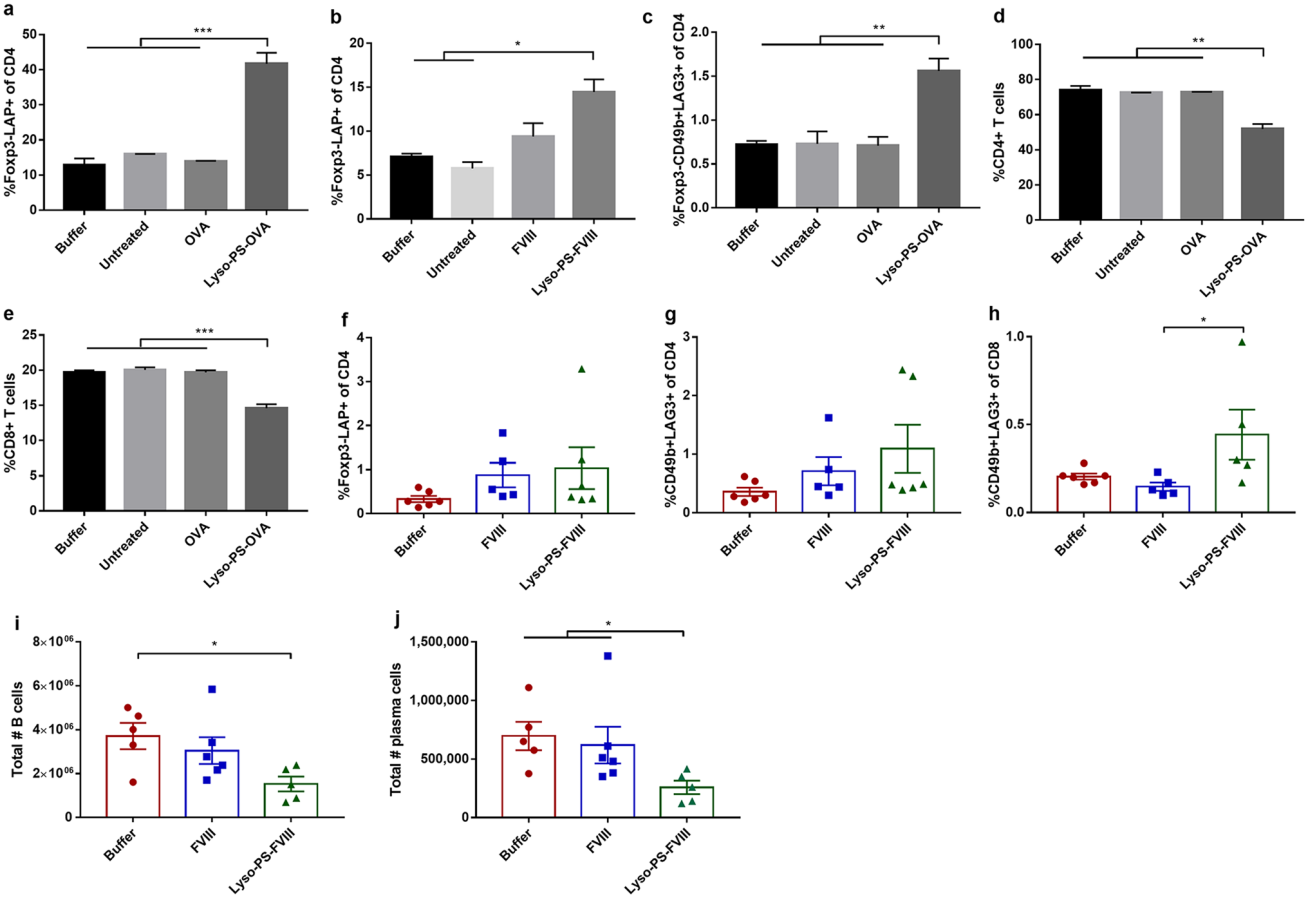
Cellular mechanisms of tolerogenic lipid particles. Expansion of LAP+ T_regs_ (**a**,**b**) and Tr1 (**c**) following in vitro treatment with tolerogenic lipid particles, which led to the suppression of CD4+ (**d**) and CD8+ T cells (**e**). Data are represented as mean ± SD. Representative graphs from 2 independent experiments was shown. (**f**–**h**) Frequency of LAP+ T_regs_, Tr1, and CD8+ T_regs_ co-expressing LAG3 and CD49b, respectively, in the MLN of animals receiving tolerogenic lipid particles. (**i**,**j**) Absolute number of B cells and antibody-producing plasma cells, respectively, in the MLN of animals receiving tolerogenic lipid particles. Data are represented as mean ± SEM. Statistical significance was denoted by P < 0.05 (*) by a one-way ANOVA test followed by Tukey’s post-hoc analysis on log-transformed data.

Studies aiming at elucidating the cellular mechanism of Lyso-PS-mediated oral tolerance were also conducted. Several classes of T_regs_ responsible for oral tolerance induction that have been widely characterized include Foxp3+ T_regs_, LAP+ T_regs_, and type 1 regulatory T cells (Tr1)^24^. Foxp3+ T_regs_ express CD4, CD25, and other markers on the cell surface, as well as Foxp3 in the nucleus. They secret suppressive cytokines such as IL-10, TGF-β, and IL-35, or granzyme to directly or indirectly inhibit effector T cells^41^. On the other hand, LAP+ T_regs_ specifically express latency-associated peptide (LAP) on their cell surface, which is an N-terminal propeptide of TGF-β and noncovalently associated with TGF-β to form inactive latent TGF-β complex^42^. As the name suggests, this class of T_regs_ secretes high levels of TGF-β as the main mechanism of action and small amounts of IL-10 and IL-4^41^. The signature combination surface markers of Tr1 are lymphocyte-activation gene 3 (LAG3) and CD49b. This class of T_regs_ is typically characterized by the production of IL-10 as the major component for their function and differentiation^43,44^. Besides IL-10, Tr1 also secrete TGF-β upon activation for their regulatory functions^41^. By exposing naïve immune cells isolated from the MLN of Swiss Webster mice to different formulations with OVA as the antigen, a significant increase in the percentage of LAP+ T_regs_ was observed in Lyso-PS treated group compared to other treatments (Fig. 3a), although a prominent change in the expansion of Foxp3+ T_regs_ was not detected. In a separate experiment in which CD11c+ DCs were isolated from the MLNs of naïve HA mice, exposed to formulations, and co-cultured with FVIII-reactive CD4+ T cells collected from FVIII-immunized mice, similar findings were consistently observed (Fig. 3b). This suggests that Lyso-PS promotes antigen-specific LAP+ T_regs_ through interaction with DCs and this can be extended and applied to multiple proteins. Besides LAP+ T_regs_, a significant induction of Tr1, which is another type of T_regs_ orchestrating oral tolerance induction^24^, was also observed in Lyso-PS treated groups compared to other treatments (Fig. 3c). Consistent with the increase in T_reg_ expansion, a decline in the percentage of live CD4+ and CD8+ T cells was detected in the culture (Fig. 3d,e). This indicates that upon the exposure to Lyso-PS nanoparticles, DCs signal naïve T cells to differentiate and proliferate into LAP+ T_regs_ and Tr1. These T_regs_ in turn suppress the activation, proliferation, and differentiation of CD4+ T cells and CD8+ T cells into effector T cells^41,45,46^. Mechanistically, the production of T_regs_ induces tolerance by directly inhibiting effector T cells and differentiation of B cells to memory B cells and antibody-producing plasma cells^47–49^. To evaluate the downstream effects of Lyso-PS on the B cell populations producing antibodies, a follow-up study was conducted in HA mice following the general oral immunization protocol shown in Fig. 1a. The results showed that mice receiving Lyso-PS-FVIII as pre-immunization had elevated frequency of LAP+ T_regs_, Tr1, and CD8+ T_regs_ co-expressing LAG3 and CD49b in the gut draining lymph node following FVIII re-challenge, although statistical significance was not observed for LAP+ T_regs_ and Tr1 (Fig. 3f–h). Co-expression of LAG3 and CD49b is a signature marker of IL-10-producing T cell lineages for both human and mouse CD4+ (Tr1) and CD8+ T_regs_^43,44^_,_ and the elevation of multiple T_regs_ subsets can synergistically act to suppress effector cells’ functions in an immunologically significant manner. Correspondingly, a significantly lower number of B cells and plasma cells were detected in the MLNs of Lyso-PS-FVIII treated animals compared to those receiving free FVIII and buffer (Fig. 3i,j). A lower frequency of B cells and plasma cells in Lyso-PS-FVIII treatment was observed than free FVIII and buffer, although not as drastic as the number of cells. This is likely because the animals were given a series of re-challenge injections, leading to a proportional activation and proliferation of effector T cells, B cells, and plasma cells in buffer and FVIII groups but not Lyso-PS-FVIII. As a result, a significant increase in cell numbers were rather observed in these two treatments.

## Discussions

On the GALT of the GI tract, M cells are a special type of epithelial cells that play an important role on the sampling, uptake, and transport of macromolecules and particulate antigens across the intestinal wall to the underlying immune cells residing beneath the M cell basolateral membrane^50,51^. The endocytosis and transcytosis of antigens by M cells are therefore crucial for the initiation of efficient immune responses and immune surveillance. Interestingly, M cells were demonstrated to predominantly express SR-B1, clusterin, and annexin V compared to the villus epithelium^18^. SR-B1 is a PS receptor that was shown to involve in the phagocytosis of PS-expressing apoptotic spermatogenic cells by Sertoli cells, as well as PS-containing liposomes^21,22^. Clusterin is a lipid transport protein that can facilitate the phagocytosis of apoptotic cells and participates in immune regulation^19^. Lastly, annexin V is a cellular protein that binds to PS with high affinity and the roles of annexin family in membrane trafficking, endocytosis, and phagocytosis have been reported^20^. Having shown that Lyso-PS nanoparticles (as well as double-chain PS) are stable in the GI tract, it is likely that the differential M cell-mediated endocytosis and transcytosis is a means of preferential uptake of Lyso-PS nanoparticles over PS nanoparticles across the epithelial barrier. After exiting M cells, Lyso-PS nanoparticles and their cargoes can be recognized and processed by various immune cell populations that expressed TIM-4, including tolerogenic CD103+ DCs, to induce effective oral tolerance^52^ through the production of antigen-specific T_regs_ and subsequent downregulation of effector T cells, B cells, and plasma cells. Alternatively, it was recently demonstrated that a population of long-lived macrophages expressing TIM-4, CD4, and CX3CR1^hi^ also resides in the murine intestinal mucosa^53^. As CX3CR1^hi^ macrophages were shown to be involved in oral tolerance induction through antigen transfer to migratory CD103+ DCs in a connexin-43 (gap junction)-dependent manner^54^, the potential role of TIM-4+ CX3CR1^hi^ macrophages to function in the uptake from the intestine and processing of Lyso-PS nanoparticles for tolerance induction is also being considered. Other cellular and molecular mechanisms can likewise be operative, and further studies are in progress to investigate the role of additional APCs (such as macrophages) and receptors (aryl hydrocarbon receptor) in Lyso-PS-mediated oral tolerance.

Overall, a reverse vaccination strategy using a nanoparticle platform was developed. This platform employs the pre-exposure of proteins in the presence of Lyso-PS nanoparticles to develop an individual’s immune tolerance towards those pre-exposed proteins, thus preventing immunogenicity upon protein-based treatments. As the risk of anti-drug antibody development is much higher during the first 20 exposures of replacement therapy for conditions such as HA^55^, this approach can supplement replacement therapy during initial exposures to effectively prevent inhibitory titers and subsequent clinical complications. Additionally, this oral immunotherapy can offer feasibility in preventing immunogenicity of transgene products, thereby opening avenues for safer and more effective gene therapies. Currently, several tolerance induction strategies are available such as polystyrene and PLGA microparticles, erythrocyte engineering, and liver targeting^56^ ^references^ ^therein^. However, our platform offers advantages over aforementioned technologies including an established regulatory path and a user-friendly oral route of administration that does not require alteration of protein antigens. Furthermore, oral consumption of PS is already widely used to reduce the risk of dementia in the elderly, and PS-based nanoparticles are amenable for (i) association and encapsulation of a wide range of proteins as well as (ii) dosage form processing conditions such as lyophilization and pediatric oral suspensions. Therefore, it is an attractive and promising approach to prevent and possibly reverse immunogenicity of a broad range of life-saving protein therapies, autoimmune diseases, and allergies.

## Materials and methods

### Materials

Brain PS, 18:1 PS, 18:1 Lyso-PS and 1,2-dimyrisotyl-sn-glycero-3-phosphocholine (DMPC) were purchased from Avanti Polar Lipids (Alabaster, AL). All recombinant FVIII products were generous gifts from the Western New York BloodCare (Buffalo, NY). Endograde Ovalbumin was purchased from BioVendor LLC (Asheville, NC) and GAA was purchased from Creative Biomart Inc (Shirley, NY). All solvents and buffer salts were obtained from Fisher Scientific (Fairlawn, NJ). PSvue 550 and ICG was obtained from Molecular Targeting Technologies, Inc. (West Chester, PA) and MP Biomedicals (Santa Ana, CA), respectively. Mouse CD11c MicroBeads UltraPure and CD4+ T Cell Isolation Kit were purchased from Miltenyi Biotec, (Bergisch Gladbach, Germany). Anti-Ovalbumin IgG1 ELISA Kit was acquired from Cayman Chemical (Ann Arbor, MI) and Endosafe Endochrome-K^®^ Kit was purchased from Charles River Laboratories (Charleston, SC). Antibodies used in staining for flow cytometry analysis were obtained from eBioscience, Inc. (San Diego, CA), BioLegend (San Diego, CA), BD Biosciences (San Diego, CA) and Tonbo Biosciences (San Diego, CA).

### Animals

A colony of hemophilic mice with a targeted deletion in exon 16 of the FVIII gene, termed HA mice, was established and maintained in our facility. These mice lack the ability to produce FVIII and therefore represent a severe HA mouse model. A colony of B6.129 6^neo^/6^neo^ GAA^−/−^ mice that is homozygous for the knockout was also maintained and bred onsite. GAA^−/−^ mice were originally bred by Raben et al.^57^. Swiss Webster mice were purchased from Charles River Laboratories (Charleston, SC). All animal experiments were conducted in strict accordance and under approval from the Institutional Animal Care and Use Committee (IACUC) at University at Buffalo, The State University of New York. Experimental procedures were performed as recommended by ARRIVE guidelines. Prior to injections, all formulations were confirmed to contain endotoxin levels less than 0.05 EU using Endosafe Endochrome-K endotoxin assay kit (Charles River Laboratories, Charleston, SC).

### Preparation of nanoparticles

Lyso-PS nanoparticles were prepared at a 30:70 molar ratio of 18:1 Lyso-PS to DMPC using the thin film method as previously described^58^. The film was then rehydrated in appropriate buffer (Tris buffer, 150 mM sodium chloride, 25 mM Tris, pH 7.0 for FVIII and OVA, or phosphate buffered saline pH 4.5 for GAA) and extruded multiple times through a double-stacked polycarbonate membrane of pore size 200 nm using a high-pressure extruder. Final concentration of the nanoparticle preparations was confirmed via a standard phosphate assay^59^. The protein to lipid molar ratio was maintained at 1:10,000 for all antigens. Association of antigens to Lyso-PS nanoparticles was achieved using the trigger-loading method by incubating the formulations at 37 °C for 30 min. This thermal stress allows for a controlled unfolding of FVIII, OVA, and GAA to promote their association with Lyso-PS nanoparticles while preserving the three-dimensional structure and biological activity^60^. The proteins are associated with the nanoparticles and are stabilized by both hydrophobic and hydrophilic interaction. The extent of these interactions depends on the structure of each antigen. PS nanoparticles containing brain PS and 18:1 PS were also prepared in a similar manner to Lyso-PS nanoparticles.

### Immunogenicity studies

#### FVIII

HA mice (n = 7/group) were continuously pre-treated with 0.4 μg of FVIII via oral gavage in the presence and absence of PS or Lyso-PS nanoparticles, or buffer as a control, once a week for 9 weeks. At the beginning of week 6, mice were also re-challenged weekly with 0.4 μg of free FVIII intravenously via the tail vein 24 h after the oral immunization for four weeks. Two weeks after the last re-challenge, all mice were sacrificed and plasma was collected via cardiac puncture in 10% *v/v* acid citrate dextrose (ACD) solution for inhibitor analysis.

#### OVA

Swiss Webster mice (n = 8/group) were continuously exposed to weekly oral immunizations of 1 µg OVA in the presence and absence of PS or Lyso-PS nanoparticles, or buffer, via oral gavage for 9 weeks. On week 6, all mice also received four weekly subcutaneous re-challenge injections with 2 µg OVA 24 h after oral immunization. Two weeks after the last re-challenge, all animals were sacrificed and plasma was collected in 10% v/v ACD solution for anti-OVA antibody analysis.

### Antigen specificity study

B6.129 GAA^−/−^ mice (n = 12/group) were orally immunized weekly for 9 consecutive weeks with 1 μg GAA in the presence or absence of Lyso-PS nanoparticles, or buffer as a control. Starting at week 6, each treatment group was divided in half (n = 6/group), with half being re-challenged with 1 μg free GAA subcutaneously, while the other half received 1 μg free OVA subcutaneously. Two weeks after the last re-challenge injection, mice were sacrificed and plasma was collected in 10% v/v ACD solution for anti-GAA antibody and anti-OVA antibody analysis.

### Determination of anti-drug antibodies

Plasma samples were collected and analyzed for the presence of anti-drug antibody development. Inhibitors against FVIII were quantified using an activated partial thromboplastin time (aPTT) assay following Nijmegen’s modified Bethesda assay, and expressed in Bethesda Units (BU/mL) as described previously^61,62^. Anti-GAA antibodies were evaluated using the Frey method and an ELISA developed in-house^13^. The concentration of Anti-OVA antibodies (µg/mL) were analyzed using the Anti-Ovalbumin IgG1 ELISA Kit (Cayman Chemical, Ann Arbor, MI). The antibody titers of some animals (7 out of 96 total, that was not specific to any particular group or treatment) were not reported as the result of unexpected animal loss or outlier identification. Detection of outliers in all treatment groups was performed using Grubb’s test with alpha set to 0.05.

### Particle size measurement

Particle sizes were determined by dynamic light scattering using a Particle Sizer and Zeta Potential Analyzer (Brookhaven's NanoBrook Omni, Holtsville, NY). Samples were allowed to equilibrate for 60 s prior to each run. Measurements were performed at 25 °C with the duration of 100 s for a total of three measurements per run.

### Measurement of PS exposure on the surface of nanoparticles

A titration study using PSvue 550 as the fluorescence probe was conducted to evaluate the exposure of PS and Lyso-PS on the surface of PS-based nanoparticles. The final concentration of PSvue was maintained at 1 μM while the concentration of all nanoparticle formulations was ranging from 0 to 280 μM. Immediately after the addition of PSvue into the formulations, samples were excited at 550 nm and emission intensity was measured at 610 nm using a SpectraMax i3 Multi-Mode Microplate Reader (Molecular Device, Sunnyvale, CA). Fluorescence intensity was normalized using the emission intensity of PSvue alone in the absence lipid. Changes in fluorescence intensity were plotted as a function of total lipid concentration and fitted in GraphPad Prism software using a single site-total binding model with nonlinear least squares fitting to evaluate for PS surface exposure.

### Stability and disposition of Lyso-PS nanoparticles in GI tract following oral administration

To track the nanoparticles along the GI tract, we associated ICG with Lyso-PS nanoparticles by adding this probe into the lipid mixture prior to the thin lipid film preparation as described previously^39^. Once nanoparticles self-assemble, ICG binds to and embedded completely within the hydrophobic domain of lipid membrane. The molar ratio of ICG to lipid was maintained at 1:250 to achieve the optimal ICG fluorescence intensity with minimal fluorescence quenching^39,40^. Mice were divided into two treatment groups receiving a single oral gavage of either aqueous ICG solution or ICG encapsulated in Lyso-PS nanoparticles at the ICG dose of 50 μg/kg. At 5 min, 1 h, and 3 h post oral administration, mice’s GI tracts were isolated for detailed organ imaging using FMT 2000 In Vivo Imaging System (Perkin Elmer, Waltham, MA).

### Role of regulatory T cells in Lyso-PS-mediated oral tolerance

A single cell suspension from the MLNs of naïve Swiss Webster mice was prepared by digesting the organs with 1 mg/mL of Collagenase D, 7.5 µg/ml of DNase I, and 25 mM HEPES in the presence of Penicillin/Streptomycin for 30 min at 37 °C in 5% CO_2_ prior to homogenization. The cells were then exposed to different formulations including buffer, OVA, or Lyso-PS-OVA at the OVA dose of 2 µg. Naïve untreated cells were used as the negative control. After 48 h, cells were harvested and stained with viability dye (Ghost Dye™ Violet 450), CD8-PE-Cy7 (2.43, Tonbo Biosciences), CD4-FITC (RM4-5), CD49b-PE (HMa2), LAG3-APC (C9B7W), Foxp3-Alexa Fluor 700 (FJK-16s, eBioscience), and LAP-PerCp-Cy5.5 (TW7-16B4, BioLegend). All flow cytometry results were obtained using a BD LSRFortessa flow cytometer (Pittsburgh, PA) and data was analyzed with FlowJo v10 (Ashland, OR).

To further evaluate for the role of DCs in Lyso-PS-mediated oral tolerance induction, naïve CD11c+ DCs were isolated from the MLNs of HA mice using the CD11c MicroBeads UltraPure kit (Miltenyi Biotec, Germany) and exposed to different formulations including buffer, free FVIII, and Lyso-PS-FVIII for 24 h. At the same time, another set of HA mice were given 2 weekly subcutaneous injections with high dose of free FVIII (2 μg) to generate FVIII-reactive T cells. Three days after the last injections, CD4+ T cells were isolated and pooled from the spleen and inguinal lymph nodes that drain the injection sites, enriched using a CD4+ T Cell Isolation Kit (Miltenyi Biotec), and co-cultured with DCs for another 3 days prior to analysis using flow cytometry. To determine the frequency of LAP+ T_regs_, cells were stained with viability dye (eFluor 450), CD4-FITC (RM4-5), Foxp3-PE (FJK-16s, eBioscience), CD3-APC (17A2, Tonbo Biosciences), and LAP-PerCP-Cy5.5 (TW7-16B4, BioLegend).

### Effect of Lyso-PS on antibody-producing cells

HA mice were given continuous oral immunization, followed by re-challenge injections as described in the “Immunogenicity Studies”. Two weeks after the last re-challenge, MLNs from all animals were isolated and stained for surface B220 (RA3-6B2, APC) and CD138 (281-2, BV421, BD Bioscience) to evaluate for the effect of Lyso-PS on B cells and plasma cells.

### Statistical analysis

All statistical analyses were performed using GraphPad Prism (GraphPad Software Inc, La Jolla, CA) version 7.0. One-way or two-way ANOVA followed by Tukey’s post-hoc analysis on the original or log-transformed values were performed to detect significant differences (*P* < 0.05) as indicated.

## Data Availability

The data that supports the findings of this manuscript are available from the corresponding author upon request.
